# A Comparative Analysis of the Chloroplast Genomes of Three *Lonicera* Medicinal Plants

**DOI:** 10.3390/genes14030548

**Published:** 2023-02-22

**Authors:** Chenju Yang, Ni Zhang, Shaoxiong Wu, Chunyan Jiang, Lian Xie, Feng Yang, Zhengwen Yu

**Affiliations:** 1 School of Life Sciences, Guizhou Normal University, Guiyang 550025, China; 2 State Key Laboratory of Functions and Applications of Medicinal Plants, Guizhou Medical University, Guiyang 550014, China; 3 The Key Laboratory of Chemistry for Natural Products of Guizhou Province and Chinese Academy of Sciences/Guizhou Provincal Engineering Research Center for Natural Drugs, Guiyang 550014, China

**Keywords:** traditional Chinese medicine (TCM), complete chloroplast genome, phylogenetic, chromatographic fingerprint, quality marker (Q-Marker)

## Abstract

Both Lonicerae japonicae flos and Lonicerae similis flos are important components in traditional Chinese medicine (TCM) with precious medicinal value. However, the absence of studies on their chloroplast genomes and chromatography has considerably hindered the study of their evolutionary and phylogenetic relationships. In this study, the complete chloroplast (cp) genomes of *Lonicera acuminata* Wall. and *Lonicera similis* Hemsl. were sequenced using the Illumina sequencing platform and compared with that of *Lonicera japonica* Thunb., which has been previously reported. Furthermore, the chromatographic fingerprints of the three plants were constructed using HPLC and the content of quality marker (Q-Marker) was calculated. The annotation results showed that the two chloroplast genomes were typical quadripartite structures with lengths of 155,330 bp (*L. acuminata*) and 155,207 bp (*L. similis*). A total of 126 different genes were annotated, containing 82 protein-coding genes, 36 tRNA genes, and 8 rRNA genes. The expansion and contraction of the inverted repeat (IR) regions suggested that the boundary regions of IR/SC were comparatively conserved in the three species, and six regions (trnH-GUG-psbA, rps2-rpoC2, rbcL-psaI, trnN-GUU-ndhF, rps15-ycf1, and infA) with nucleotide diversity values (Pi) of variable sites higher than 1% were identified. Phylogenetic relation indicated that *L. similis* had a closer genetic relationship with *L. japonica* than *L. acuminata*. Additionally, the chromatographic fingerprints showed that the characteristic peaks of the three medicinal plants were similar, including Neochlorogenic acid, Chlorogenic acid, 4-Dicaffeoylquinic acid, Sweroside, Secoxyloganin, Luteoloside, Isochlorogenic acid A, Isochlorogenic acid B, and Isochlorogenic acid C. The content of chlorogenic acid and total phenolic acid in *L. acuminata* (7.4633 ± 0.4461%, 14.8953 ± 0.0728%) and *L. similis* (14.1055 ± 0.2566%, 21.9782 ± 0.1331%) was much higher than that of *L. japonica* (3.9729 ± 0.0928%, 6.0964 ± 0.1228%), respectively. This study provides appropriate information for species identification, phylogeny, quality assessment, and rational use of three medicinal plants of the genus *Lonicera*.

## 1. Introduction

Lonicerae japonicae flos, a traditional Chinese medicine (TCM), has been extensively used for several thousand years in China [1,2,3,4]. In the 2020 edition of the *Pharmacopoeia of the People’s Republic of China* (Chinese Pharmacopeia), Lonicerae japonicae flos is defined as the dried flower buds or initial flowers of *L. japonica* Thunb., which belongs to Caprifoliaceae [5]. As a famous TCM, Lonicerae japonicae flos has antiviral, anti-inflammatory, and antibacterial activity, and pharmacological studies have shown it is effective in the treatment of inflammation and infectious diseases [3,6,7,8,9]. Significantly, Lianhua Qingwen Capsule/Granule, Jinhua Qinggan Granule, and Shuanghuanglian Oral Liquid, three patented Chinese medicines containing Lonicerae japonicae flos, are recommended as the effective “Chinese Solution” to combat COVID-19 [10,11,12,13,14,15,16]. Recently, Lonicerae japonicae flos has been widely cultivated in Shandong and Henan provinces in China [17], though it is still far from meeting the market demand [7].

Lonicerae similis flos and Lonicerae japonicae flos belong to the same family. In the 2010 edition of the *Standard of Traditional Chinese Medicine in Sichuan Province*, Lonicerae similis flos consists of the dried flower buds or initial flowers of *L. acuminata* Wall. or *L. similis* Hemsl. [18,19,20]. Lonicerae similis flos is mainly distributed in Sichuan Province and widely used on account of the abundance of resources [20]. Similarly, Lonicerae similis flos is effective in clearing heat, removing toxins, and dispelling wind and heat [17].

Although Lonicerae japonicae flos and Lonicerae similis flos were divided into two herbs according to their origins, they still have close proximity in plant species, appearances, and functions, together with traditional applications and great homogeneity in light of their medicinal uses [2,3,17,20]. Meanwhile, they also contain an array of identical chemical components, including Neochlorogenic acid, Chlorogenic acid, Isochlorogenic acid A, Isochlorogenic acid B, Isochlorogenic acid C, etc. [17,20,21]. Moreover, it is commonly adulterated with Lonicerae similis flos, motivated by economic interests on the basis of higher prices of Lonicerae japonicae flos [22]. Therefore, the close relationship of Lonicerae japonicae flos and Lonicerae similis flos in terms of morphology, chemical composition, potency, and therapeutic properties is controversial, especially concerning their quality standards and interchangeability. 

It is difficult to distinguish Lonicerae japonicae flos and Lonicerae similis flos based on the similarity of their morphology, thus causing a mixture of the two species, which to some extent affects the safety and efficacy of the medicines base on Lonicerae japonicae flos. However, previous studies have mainly focused on morphology, chemical composition, and pharmacological activity; morphological studies are still the main means of species identification, thus causing inaccurate interspecific identification of the species within the genus [2,3,17,20,23,24]. The structure of the plant cp genome is a typical quadripartite structure, including two copies of IR regions which are segregated by a large single-copy (LSC) region and a small single-copy (SSC) region [25,26,27]. The sizes of the cp genomes of different species vary depending on IR contraction or expansion [28,29]. As next-generation sequencing technologies are progressing rapidly, the cost of obtaining genomes is now lower and faster than traditional Sanger sequencing techniques [30]. As a result, cp genome-scale data are increasingly being used to infer phylogenetic relationships at elevated levels of taxonomy, and great progress has been made even in lower taxonomic groups [31]. The cp genome has haploid genetics, conservative structuring, a comparatively small genome, and a sluggish burst rate of mutations, which makes it an ideally suited model for the molecular identification of species, for studies of genetic diversity, and for revealing phylogenetic relationships [32,33,34]. Furthermore, chromatographic fingerprint and Q-Marker content have become important indicators for the identification and quality evaluation of TCM [5,35,36], which will greatly increase the accuracy of interspecific identification and provide a reliable basis for the identification and quality evaluation of the herbs in question.

In the present study, we aimed to obtain the cp DNA sequences of *L. acuminata* and *L. similis* by using high-throughput Illumina pair-end sequencing data, and compared the two cp genomes with previously reported studies, gene number, type, and repeat sequences of Lonicerae japonicae flos. Moreover, phylogenetic analysis was performed by using whole cp genomes of 25 plants. Finally, the chromatographic fingerprints of the three plants were constructed and the content of quality marker (Q-Marker) was calculated using HPLC. This study provides a valuable resource for species identification, quality evaluation, and genetic improvement of the genus *Lonicera*.

## 2. Results

### 2.1. Cp Genome Organizations

**Figure 1:**
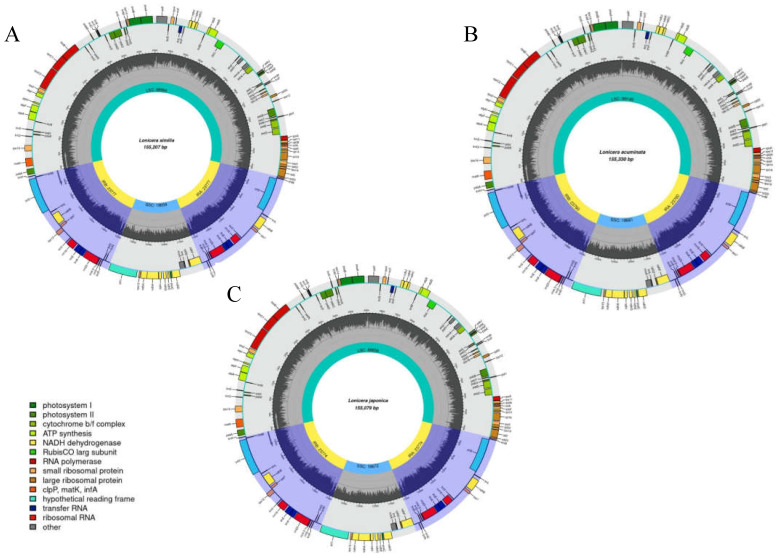
Gene maps of the three *Lonicera* chloroplast genomes. (**A**) *L. similis*; (**B**) *L. acuminata*; (**C**) *L. japonica*. Small single copy (SSC), large single copy (LSC), and inverted repeats (IRA, IRB) are indicated. The black of the inner circle indicates GC content, and dark gray indicates AT content.

We used high-throughput Illumina paired-end sequencing data to acquire cp DNA sequences of *L. acuminata* and *L. similis*, and the annotated, complete chloroplast genomes of the two species were then deposited in GenBank under accession numbers MZ901373 and MZ241297, respectively. Three complete cp genome sequences, including the two *Lonicera* species sequenced in this study and the *L. japonica* accession from GenBank, were combined for integrated analysis. The genomes ranged in size from 155,207 (*L. similis*) to 155,330 bp (*L. acuminata*) (Figure 1). The genomes of these species have a typical quadripartite structure, with two IRs (23,760–23,777) separated by the LSC (88,859–89,149) and SSC (18,659–18,672) regions. The GC content of the *L. japonica*, *L. similis,* and *L. acuminata* cp genomes is 38.58%, 38.59%, and 38.55%, respectively (Table 1); these three species showed similar GC levels (~39%). *Lonicera* cp genomes displayed analogous gene content and order, containing 126 genes, 82 protein-coding genes, 36 tRNA genes, and 8 rRNA genes.

### 2.2. Repeat and Simple Sequence Repeat (SSR) Analyses

**Figure 2:**
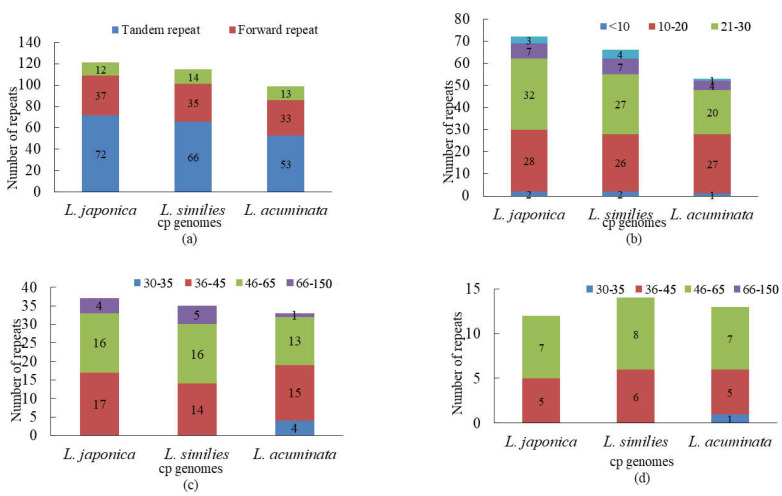
Repeat sequences analysis of three cp genomes. (**a**) Repeat types in three cp genomes; (**b**) tandem repeats in three cp genomes; (**c**) forward repeats in three cp genomes; (**d**) palindromic repeats in three cp genomes. In (**a**), different colors show different repeat types; in (**b**–**d**), different colors show different lengths. The ordinate represents the number of repeats; the abscissa represents three cp genomes.

A comparative analysis of the repeats in the three *Lonicera* cp genomes suggested that the three types of repeats (tandem, forward, and palindromic repeats) were similar in number and distribution (Figure 2a). *L. japonica* presented the highest frequency of tandem repeats (72) and forward repeats (37), while *L. acuminata* had the lowest with (53) and (33), respectively. Palindromic repeats did not differ much between *L. japonica* (12), *L. similis* (14), and *L. acuminata* (13). The tandem repeats in the three *Lonicera* cp genomes ranged from 10 to 30 bp in length (Figure 2b), while the forward and palindromic repeats were mostly between 36 and 65 bp in length (Figure 2c,d).

**Figure 3:**
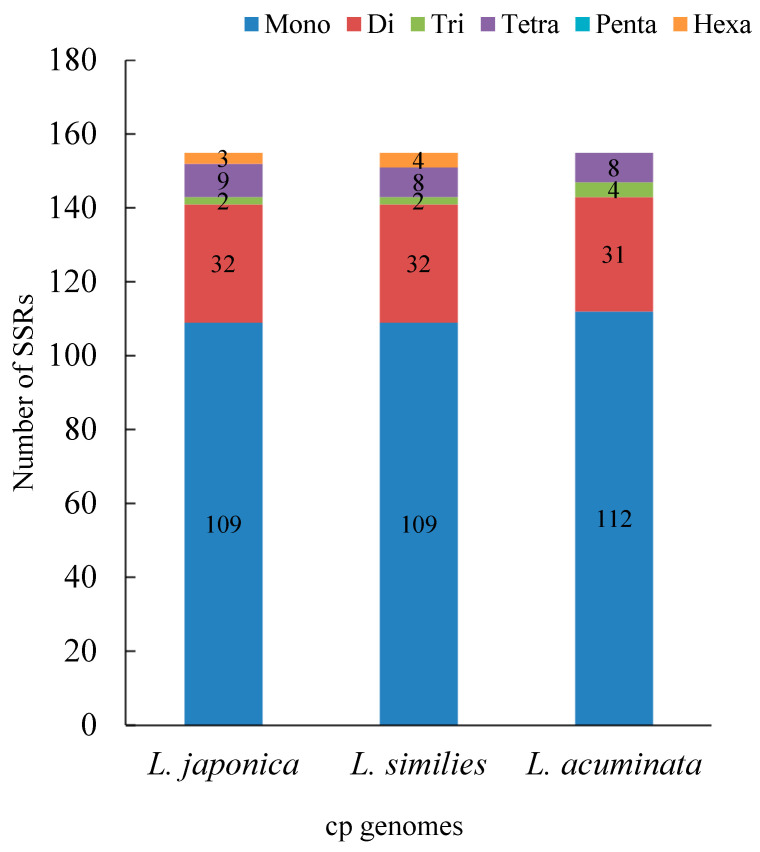
SSR analysis of three cp genomes. The ordinate represents the number of SSRs; the abscissa represents three cp genomes.

The cp genome is uniparentally inherited and SSRs share a high level of variation within the same species [27]. As such, they are used as molecular markers in developmental research and also help to identify species [37]. MISA detected 155 SSRs in *L. japonica*, comprising 109 mono-, 32 di-, 2 tri-, 9 tetra-, and 3 hexanucleotide repeats. Here, a grand total of 155 SSRs were detected in the *L. similis* cp genome; among these SSRs, there were 109, 32, 2, 8, and 4 for mono-, di-, tri-, tetra-, and hexa-nucleotide repeats, respectively. No pentanucleotide repeats were discovered. A total of 155 SSRs, comprising 112 mono-, 31 di-, 4 tri-, and 8 tetra-nucleotide repeats were observed in the *L. acuminata* cp genome, while no penta- and hexa-nucleotide repeats were found (Figure 3). 

SSRs of the three cp genomes were composed of mono- and di- repeat motifs. In the *L. japonica* and *L. similis* cp genomes, there are the same number of mono- repeats and di- repeats: 109 and 32, respectively. In the *L. acuminata* cp genome, the mono- repeats and di- repeats number 112 and 31, respectively. However, the number of three or more oligonucleotide repeats is comparatively low.

### 2.3. IR Expansion and Contraction

**Figure 4:**
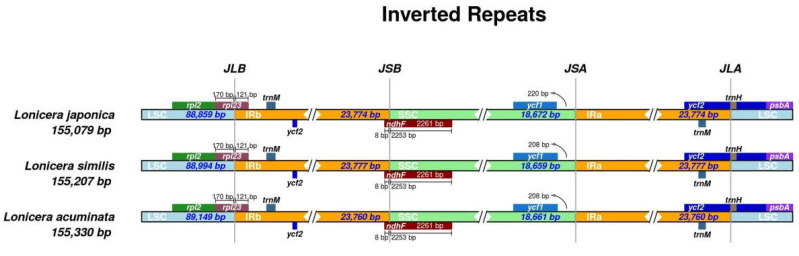
Comparative analyses of the boundary regions (LSC, SSC, and IR) among three chloroplast genomes of *Lonicera*. Gene names are indicated in boxes, and their lengths in junction sites are displayed above the boxes.

It is reported that the chloroplast genome of angiosperms is conserved in structure and size [37]; the differences in IRs may reflect phylogenetic history [38]. Here, we selected three species of the family Caprifoliaceae and compared the sizes and junctions of their LSC, SSC, and IR regions. Although the lengths of the IR regions, ranging from 23,760 bp to 23,777 bp, varied little among the three species, some differences in the IR expansions and contractions were observed. As shown in Figure 4, the rpl2 gene of all three species was located in the LSC region, the rpl23 gene was located at the LSC/IRb border expanded 121 bp into the IRb region, and the ndhF gene of the three species extended 8 bp into the IRb region. For the SSC/IRa boundaries in *L. japonica*, *L. similis*, and *L. acuminata*, the distances were 220 bp, 208 bp, and 208 bp from the ycf1 gene to the boundary, respectively (Figure 4). 

### 2.4. Sequence Divergence Analysis

**Figure 5:**
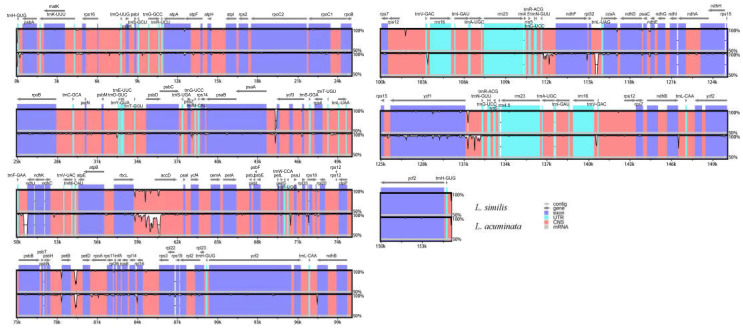
Sequence alignment of three chloroplast genomes in the Caprifoliaceae family performed with mVISTA using annotation of *L. japonica* as reference. The top arrow shows transcription direction; genome regions are color coded as exon, Untranslated Region (UTR), mRNA, and conserved non-coding sequences (CNS). The *x*-axis represents the coordinates in the cp genome while the *y*-axis represents percentage identity within 50–100%.

**Figure 6:**
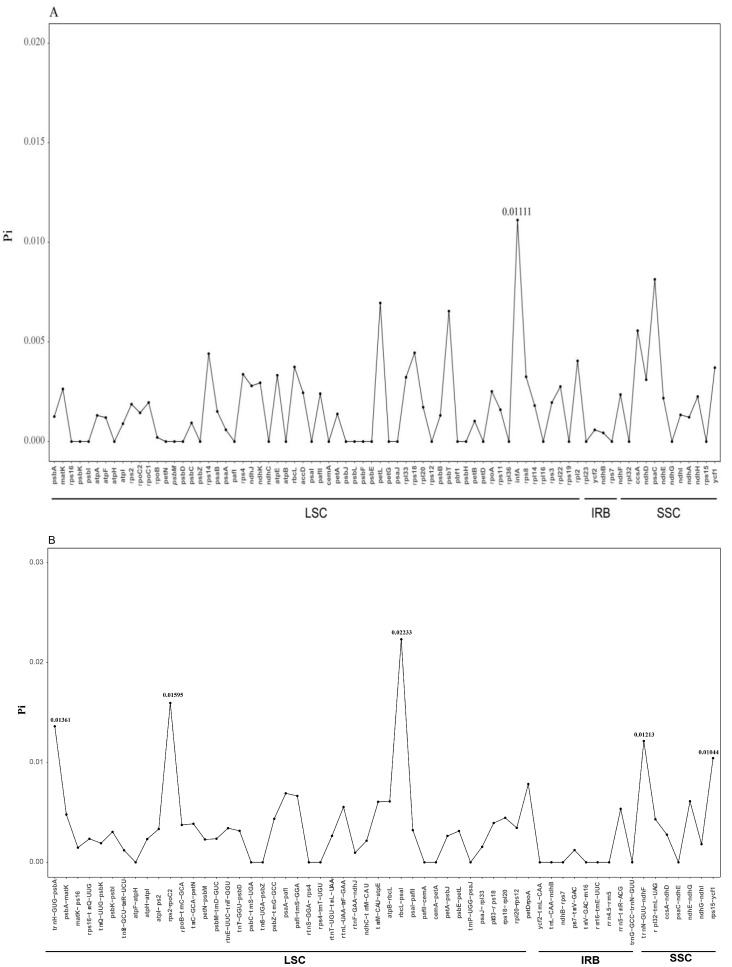
The nucleotide variability (Pi) values were compared among *L. acuminata*, *L. similis,* and *L. japonica*. (**A**) CDS region; (**B**) Intergenic spacer region.

The three complete cp genomes were compared with mVISTA using *L. japonica* as a reference. As shown in Figure 5, high sequence conservation across the three *Lonicera* cp genomes was observed, especially in gene regions. Variant loci in intergenic regions were distinguished higher than those in the gene regions. Most of the highly variable regions were in the conserved non-coding sequences (CNS) region, included rps16-trnQ, ycf1-trnN, psaA-ycf3, rbcL-accD, psaJ-clpP, and petB-petD, etc. In order to clarify the variation in the higher regions, the nucleotide diversity values (Pi) were calculated using the DnaSP v.6.10 software (Figure 6). The variation in intergenic regions ranged from 0 to 2.23%, with an average of 0.36%, which was twofold higher than that in the CDS regions (0.16% on average). Five divergent loci in intergenic regions (trnH-GUG-psbA, rps2-rpoC2, rbcL-psaI, trnN-GUU-ndhF, and rps15-ycf1) and one in the CDS regions (infA) had a Pi exceeding 1%. These six divergence hotspot regions should be applied to the development of molecular markers for phylogenetic and phylogeographic analyses, as well as plant identification of *Lonicera* species.

### 2.5. Phylogenetic Analysis

**Figure 7:**
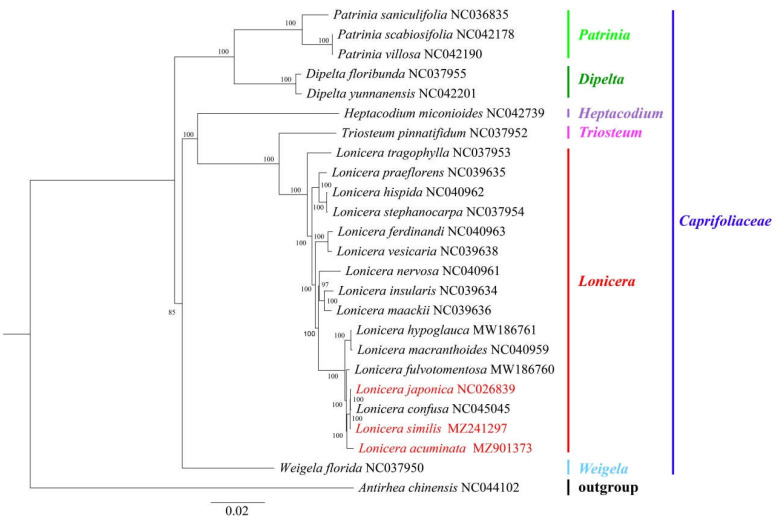
Maximum-likelihood tree based on the complete chloroplast genome sequences of 25 species. GenBank accession numbers are described in the figure. Shown next to the nodes are bootstrap support values based on 1000 replicates.

To further analyze the relationships between *L. acuminata*, *L. similis,* and *L. japonica*, 22 chloroplast genome sequences of the family Caprifoliaceae and one outgroup (*Antirhea chinensis*) were selected and downloaded from GenBank to construct phylogenetic trees with *L. acuminata* and *L. similis,* adopting the maximum-likelihood (ML) method. The ML tree based on the GTR + G + I optimal model was constructed through IQ-tree (Version 2.04) with 1000 bootstrap replicates [39]. In our study, the selected species only came from six genera of the family Caprifoliaceae. Figure 7 clearly displays that all species generated six major clades, which correspond to the six selected genera. All *Lonicera* were clustered together in one monophyletic group, between *L. acuminata*, *L. similis,* and *L. japonica* of the genus *Lonicera* and family Caprifoliaceae. *L. japonica* was clustered together with *L. confusa* and *L. similis*, which implicates that *L. similis* may have a closer genetic relationship with *L. japonica* than *L. acuminata*. 

### 2.6. Plant Morphology

It can be seen in Table 2 that the main medicinal parts (flower buds and flowers) of Lonicerae japonicae flos and Lonicerae similis flos have a fresh scent and a light and slightly bitter taste, but they differ more markedly in color, length, and upper swollen parts. In our study, *L. japonica* was yellow-white in color, *L. similis* was yellow-brown, and *L. acuminata* was yellow-green. For the medicinal parts of the three plants (flower buds and flowers), *L. similis* was the longest but shortest in upper diameter, *L. acuminata* was the longest in upper diameter, and *L. japonica* was in the middle.

### 2.7. HPLC Fingerprints and the Content of Main Q-Marker

**Figure 8:**
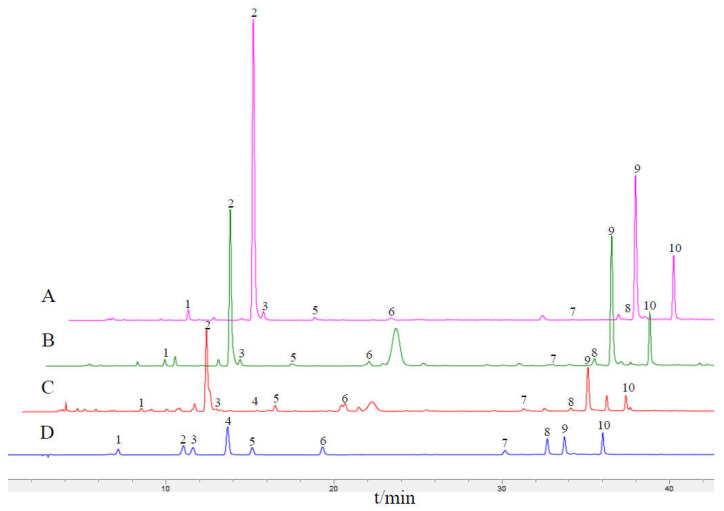
HPLC fingerprints of mixed control and sample. *L. similis* (**A**), *L. acuminata* (**B**), *L. japonica* (**C**), mixed control (**D**). Neochlorogenic acid (1), Chlorogenic acid (2), 4-Dicaffeoylquinic Acid (3), Caffeic acid (4), Sweroside (5), Secoxyloganin (6), Luteoloside (7), Isochlorogenic acid B (8), Isochlorogenic acid A (9), Isochlorogenic acid C (10).

In terms of chemical composition, HPLC fingerprints of the three species were obtained by high performance liquid chromatography (HPLC) analysis at 240 nm with reference to the *Chinese Pharmacopoeia* [5] and superimposed on the control (Figure 8). From the fingerprints, we can clearly see that the main active ingredients of Lonicerae similis flos and Lonicerae japonicae flos are basically the same, including Neochlorogenic acid, Chlorogenic acid, 4-Dicaffeoylquinic acid, Sweroside, Secoxyloganin, Luteoloside, Isochlorogenic acid A, Isochlorogenic acid B, Isochlorogenic acid C, etc., but the content of main Q-Marker differs (Table 3). The evaluation standards for *L. japonica* in the *Chinese Pharmacopoeia* [5] require it to contain not less than 1.5% chlorogenic acid, not less than 3.8% total phenolic acid, and not less than 0.050% Luteoloside. Here, the content of chlorogenic acid and total phenolic acid in *L. acuminata* (7.4633 ± 0.4461%, 14.8953 ± 0.0728%) and *L. similis* (14.1055 ± 0.2566%, 21.9782 ± 0.1331%) was much higher than that of *L. japonica* (3.9729 ± 0.0928%, 6.0964 ± 0.1228%), respectively. The content of Luteoloside in *L. similis* (0.0291 ± 0.0044%) was lower than that specified in the pharmacopoeia, but it is noteworthy that the content of chlorogenic acid, its main purgative and detoxifying component, was higher than that of all other species at 14.8953 ± 0.0728%. 

## 3. Materials and Methods

### 3.1. Plant Material and DNA Extraction

*L. similis* was collected in the town of Dangwu, Huaxi District, Guiyang City (26°23′34.49″ N, 106°35′56.89″ E, 1158 m above sea level), Guizhou Province, China, and *L. acuminata* was harvested from the village of Hekou, the town of Muxi, Muchuan County, Sichuan Province, China (28°51′7.40″ N, 103°50′55.46″ E, 1114 m altitude) by Chenju Yang. According to the macroscopic morphological characteristics of the specimen, the species was determined [40]. Total genomic DNA of two species was extracted using an E.Z.N.A^®^ plant DNA kit (FEIYANG, Guangzhou, China). For each sample a combined total of 1000 ng of DNA served as input to the DNA sample preparation. The DNA library was established utilizing the TruseqTM RNA Sample Prep Kit. Whole DNA was utilized to generate libraries of an average insert size of 400 bp. An Illumina platform was used to sequence the library preparation and obtained approximately 3 GB of 150 bp paired-end reads, which were saved in fastq format.

### 3.2. Assembly and Annotation

FastQC was used to examine the acquired raw data for analysis of quality, including single base content quality, base content mapping, GC content distributions, and quality of sequence bases. After removal of the adapter sequences, program GetOrganelle was used to assemble the filtered reads [41] using *L. japonica* (GenBank accession number: NC_026839) for the original genome reference, and the genome of the assembled chloroplast was annotated by the GeSeq online package [42]. Finally, the accurate, annotated, complete chloroplast genomes of two *Lonicera* species were deposited to GenBank with accession numbers MZ241297 and MZ901373. Here, the document we use was further annotated based on the accession number. 

### 3.3. Characterization of Repeat Sequences and SSRs

Repeat sequences, comprising the forward and palindromic, were identified by REPuter [43] with a Hamming distance set at 3 bp and a minimum repeat size set at 30 bp. Tandem repeats were analyzed using the Tandem Repeats Finder with default parameters [44]. SSRs were detected using MISA with the following thresholds [45]: eight repeat units for mono SSRs, four repeat units for di- and trinucleotide repeat SSRs, and three repeat units for tetra-, penta-, and hexanucleotide repeat SSRs.

### 3.4. Comparative Genome Analysis and Sequence Variation

The program mVISTA in the Shuffle-LAGAN mode was applied to compare the cp genomes of Lonicerae japonicae flos (*L. japonica*) and Lonicerae similis flos (*L. acuminata* and *L. similis*) [46]. The variable character for coding and non-coding region with an aligned length of more than 200 bp was obtained based on the method of Zhang et al. [47]. The nucleotide variability was calculated with DnaSP 6.10 [48]. The online software IRSCOPE (https://irscope.shinyapps.io/irapp/, accessed on 31 October 2022) was adopted to carry out boundary analysis on the IR regions of the three species [49]. 

### 3.5. Phylogenetic Analysis

The chloroplast genome sequences of 22 Caprifoliaceae family and one outgroup (*A. chinensis*) were downloaded from GenBank to build phylogenetic trees by maximum-likelihood (ML) with *L. acuminata* and *L. similis*. The ML tree was constructed using IQ-tree (Version 2.04, Trifinopoulos J., Vienna，Austria) based on the GTR + G + I optimal model with 1000 bootstrap replicates [39,50]. 

### 3.6. HPLC Analyses

The sample solutions were obtained according to the method described in *Chinese Pharmacopoeia Commission* (2020) [5]. HPLC analyses were carried out through an Agilent 1260 Infinity II series system (Agilent Technologies, Santa Clara, CA, USA) consisting of a G1311B pump, a G4212B DAD detector, and a G1329B autosampler. J&KCHEMICA C18 column (250 × 4.6 mm, 5 µm) was adopted for the analyses. The mobile phase consisted of A (acetonitrile), B (0.1% phosphoric acid solution), and C (methanol). The gradient mode was as follows: 0~10 min, 6~10% A; 10~25 min, 10~14% A; 25~35 min, 14~24% A; 35~40 min, 24~27% A; 40~45 min, 27~35% A; 45~50 min, 35~46% A; 50~55 min, 46~51% A; 55~57 min, 51~6% A; 57~70 min, 90% B; C 4% for all times. The flow rate was 1.0 mL/min. The detection wavelength was set at 240, 326, and 350 nm. The column temperature was set at 30 °C and sample volume was set at 5 µL. 

## 4. Discussion

In this study, we conducted a comprehensive comparative analysis of Lonicerae similis flos and Lonicerae japonicae flos. The cp genomes of the three species ranged in length from 155,079 (*L. japonica*) to 155,330 bp (*L. acuminata*); they had a typical quadripartite structure, usually consisting of an LSC, an SSC, and two IR regions, which is consistent with highly conserved features of the cp genome of most angiosperms [25]. Compared with previous studies [33,51,52], chloroplast genomes of *Lonicera* plants differ little in terms of genome organization, number of genes, and types. In a comparative analysis of repeat sequences in the cp genomes, *L. japonica* (a total of 121 repeats, 72 tandem, 37 forward, 12 palindromic repeats) and *L. similis* (a total of 115 repeats, 66 tandem, 35 forward, 14 palindromic repeats) repeats were similar, while *L. acuminata* (a total of 99 repeats, 53 tandem, 33 forward, 13 palindromic repeats) had shorter fragment repeats. The three cp genomes had similar repeat unit lengths, with tandem repeats ranging from 10 to 30 bp and forward and palindromic repeats ranging from 36 to 65 bp. The cp genome is uniparentally inherited and SSRs have a high level of variation within the same species [27]. As such, they are used as the molecular indicators in developmental studies and also help to identify species [37]. SSRs are commonly used as genetic markers in community genetics and evolutionary studies [31]. The most enriched were mononucleotide repeats, next to dinucleotide repeats. As a whole, tetranucleotide repeats were slightly more abundant than trinucleotide and hexanucleotide repeats, and in all three cp genomes pentanucleotide repeats were rare. The three species shared similar IR expansion and contraction, and in sequence alignments had higher variance genes in the CNS region, including rps16-trnQ, ycf1-trnN, psaA-ycf3, rbcL-accD, psaJ-clpP, and petB-petD, etc. The nucleotide diversity values (Pi) showed that six regions (trnH-GUG-psbA, rps2-rpoC2, rbcL-psaI, trnN-GUU-ndhF, rps15-ycf1, and infA) had a Pi exceeding 1%, and these six divergence hotspot regions should be useful for developing molecular markers. In the phylogenetic tree, *L. japonica* may be more closely related to *L. similis* than *L. acuminata*. Our evolutionary tree formed three main branches, which was consistent with previous research [33]. At the same time, plants in the same growing environment may be more closely related.

Despite their different origins, they have close proximity of plant species, appearances, and functions, together with traditional applications [5,19]. Studies have shown that chromatographic fingerprint analysis provides a more rational method for the identification and quality assessment of traditional Chinese herbal medicines [35,36], which can effectively identify the authenticity, merit, and demerit of traditional Chinese medicines and can increase the accuracy of species identification. In this study, we used high performance liquid chromatography (HPLC) to obtain HPLC fingerprints of three species and calculated their contents with reference to the *Chinese Pharmacopoeia* [5]. HPLC fingerprints showed that Lonicerae similis flos and Lonicerae japonicae flos shared the same characteristic peaks, except for Lonicerae similis flos where Caffeic acid was not detected (peak 4), but the contents of quality marker (Q-Marker) were different. The content of Luteoloside (0.0291 ± 0.0044%) in *L. similis* was lower than that specified in the pharmacopoeia for Lonicerae japonicae flos, but it is noteworthy that the content of chlorogenic acid and total phenolic acid in *L. acuminata* (7.4633 ± 0.4461%, 14.8953 ± 0.0728%) and *L. similis* (14.1055 ± 0.2566%, 21.9782 ± 0.1331%) was much higher than that of *L. japonica* (3.9729 ± 0.0928%, 6.0964 ± 0.1228%), respectively. Chlorogenic acid is known as “plant gold”, which has hypotensive effects, antioxidant, and antibacterial activities [53,54,55,56,57]. It is may be one of the potential active ingredients of Lonicerae japonicae flos in the treatment of COVID-19 [58]. With such high chlorogenic acid content, *L. similis* may be selected as a better genetic line for superior breeding and a better source species for chlorogenic acid. These results also provide a basis for future in-depth studies on the synthesis mechanisms of high chlorogenic acid in *L. similis*.

## 5. Conclusions

In this study, the cp genomes of Lonicerae similis flos (*L. acuminata* and *L. similis*) were sequenced and annotated through high-throughput sequencing technology. We compared the cp genomes of these three species, and bioinformatics analysis revealed that the structure and gene content of the cp genomes of these three *Lonicerae* were highly similar and conserved, suggesting a close relationship between them. We identified 155 SSR loci that could potentially be used as molecular markers to study the diversity of the genus *Lonicera.* Six different mutation hot spots (trnH-GUG-psbA, rps2-rpoC2, rbcL-psaI, trnN-GUU-ndhF, rps15-ycf1, and infA) with high nucleotide variability (Pi) could be considered as potential DNA barcodes of *Lonicera* species. In our ML tree, all *Lonicera* were clustered together in one monophyletic group and were closely related to the *Triosteum* plants. The HPLC fingerprints of Lonicerae japonicae flos and Lonicerae similis flos increased the accuracy of species identification. The content of Q-Marker showed great variation; the content of chlorogenic acid and phenolic acids (chlorogenic acid, isochlorogenic acid A, isochlorogenic acid C) in Lonicerae similis flos is much higher than that in Lonicerae japonicae flos. It is noteworthy that the content of chlorogenic acid was as high as 14.8953 ± 0.0728% in *L. similis*, which can be considered as a raw material for this compound. Therefore, it is important to explore the molecular mechanism of high chlorogenic acid in *L. similis*.

This study of the cp genomes of three *Lonicera* provides valuable information for the species, enriches cp genomic data of *Lonicera*, and gives genetic resources for further species identification and phylogenetic studies of the genus. Moreover, chromatographic fingerprint analysis is an effective supplementary method to identify three TCMs, and the differences in content of Q-marker will provide a reference for the quality evaluation and rational use of Chinese herbal medicines of the genus *Lonicera*. In future research, we will perfect the data related to the quality standard of Lonicerae similis flos to provide reference for bringing it into the pharmacology of the People’s Republic of China.

## Figures and Tables

**Table 1:** Summary of complete cp genomes of three *Lonicera* species.

Species	Genome Size(bp)	LSC Region (bp)	IR Region (bp)	SSC Region (bp)	Number of Genes	PCGs	tRNAs	rRNAs	GC Content (%)	Accession Number in Genbank
*L. japonica*	155,079	88,859	23,774	18,672	126	82	36	8	38.58	NC_026839
*L. similis*	155,207	88,994	23,777	18,659	126	82	36	8	38.59	MZ241297
*L. acuminata*	155,330	89,149	23,760	18,661	126	82	36	8	38.55	MZ901373

**Table 2:** The comparison of Botanical traits of Lonicerae japonicae flos and Lonicerae similis flos.

Botanical Traits	*L. japonica*	*L. similis*	*L. acuminata*
Color and glandular hairs	Surface yellow-white or green-white, densely pubescent	Surface yellow-green, green-brown or yellow-brown, covered with long, short coarse or glandular hairs, some glabrous	Surface yellow-green, brown-yellow, pale-purple to purplish-brown, glabrous or sparse
Shape	Rod-shaped, slightly curved, 2–3 cm long, upper diameter about 3 mm, lower 1.5 mm	Slender rod-shaped, slightly curved, 3–6 cm long, slightly inflated above, upper diameter 1.8–2 mm, lower 1.2–1.5 mm	Short rod-shaped, 1–2 cm long, distally expanded, upper diameter 1.5–3.5 mm, lower 0.6–1.5 mm
Smell and taste	Fresh scent, light and slightly bitter taste	Fresh scent, light and slightly bitter taste	Fresh scent, light and slightly bitter taste
Flower buds and flowers	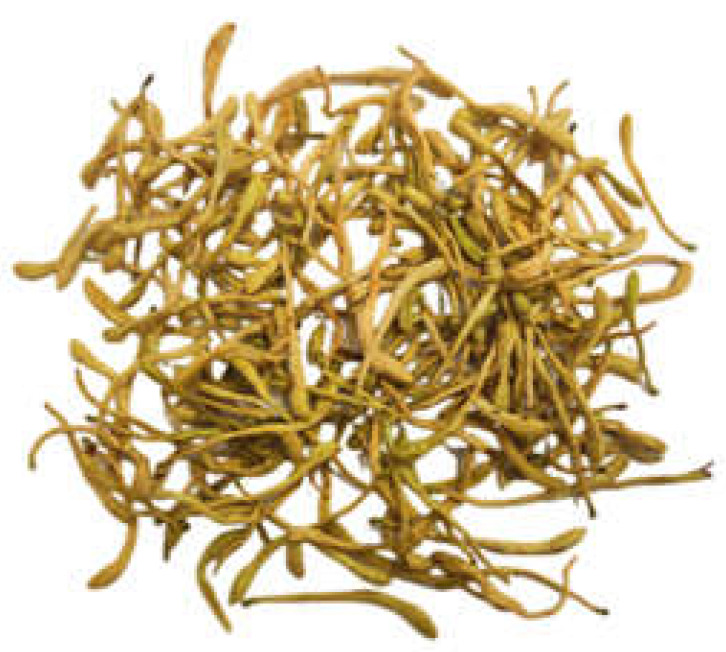	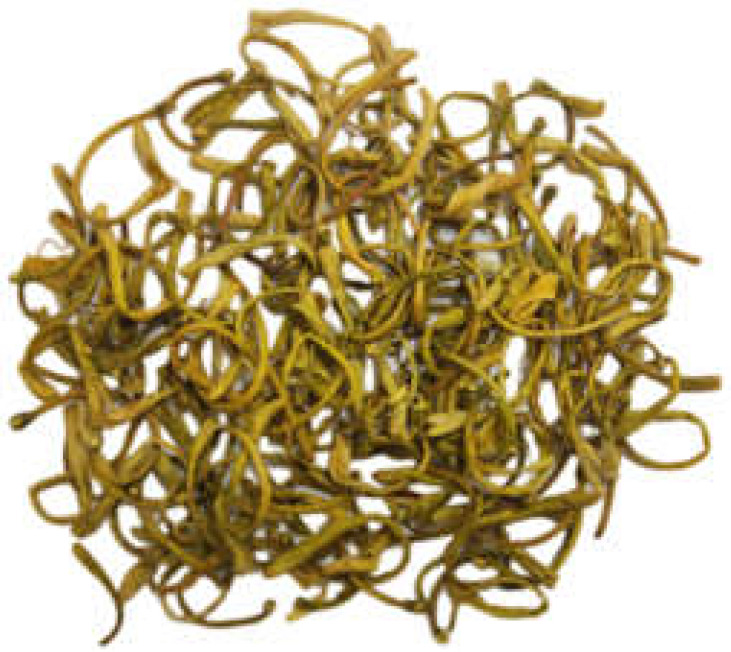	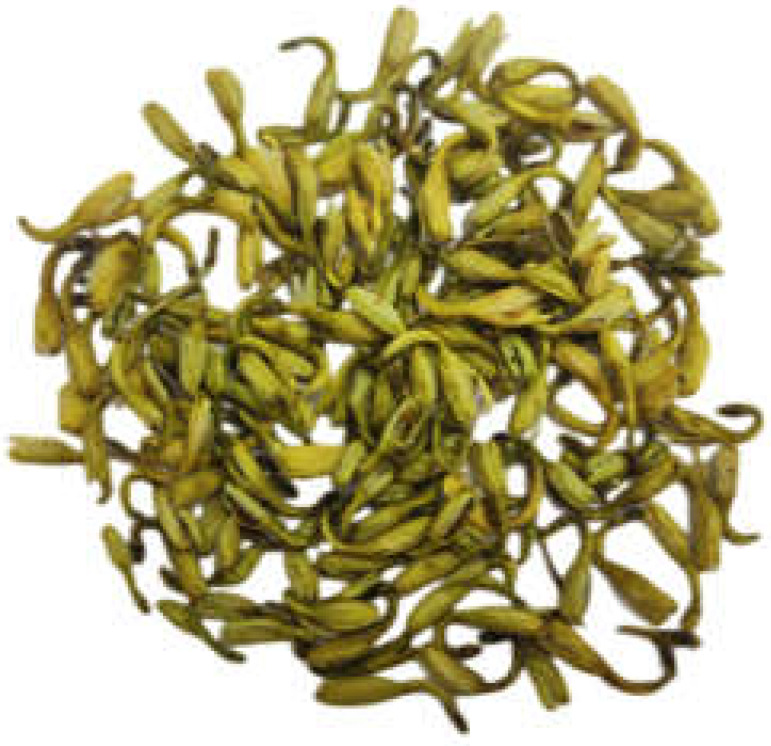
Reference	National Pharmacopoeia Committee, 2020	Si Chuan Food and Drug Administration, 2011	Si Chuan Food and Drug Administration, 2011

**Table 3:** The content of main Q-Marker in Lonicerae japonicae flos and Lonicerae similis flos (%).

Species	NA	CA	4-DA	SW	LS	IA-B	IA-A	IA-C	TPC
*L. japonica*	0.0773 ± 0.007	3.9729 ± 0.0928	0.0584 ± 0.0041	0.1835 ± 0.0086	0.0549 ± 0.0001	0.0227 ± 0.0004	1.7260 ± 0.0161	0.3975 ± 0.0461	6.0964 ± 0.1228
*L. similis*	0.3098 ± 0.0013	**14.8953 ± 0.0728**	0.2749 ± 0.0515	0.0729 ± 0.0062	0.0291 ± 0.0044	0.0603 ± 0.0014	5.4567 ± 0.0278	1.6262 ± 0.0882	21.9782 ± 0.1331
*L. acuminata*	0.2462 ± 0.0400	7.4631 ± 0.4461	0.1519 ± 0.0507	0.0852 ± 0.0011	0.2248 ± 0.0256	0.0613 ± 0.0024	5.4267 ± 0.3922	1.2156 ± 0.2027	14.1055 ± 0.2566

Neochlorogenic acid (NA), Chlorogenic acid (CA), 4-Dicaffeoylquinic Acid (4-DA), Sweroside (SW), Luteoloside (LS), Isochlorogenic acid B (IA-B), Isochlorogenic acid A (IA-A), Isochlorogenic acid C (IA-C), Total phenolic acid (TPC).

## Data Availability

The mentioned chloroplast genome data that support the findings of this study are openly available in GenBank of NCBI at https://www.ncbi.nlm.nih.gov, accessed on 18 May 2021.
